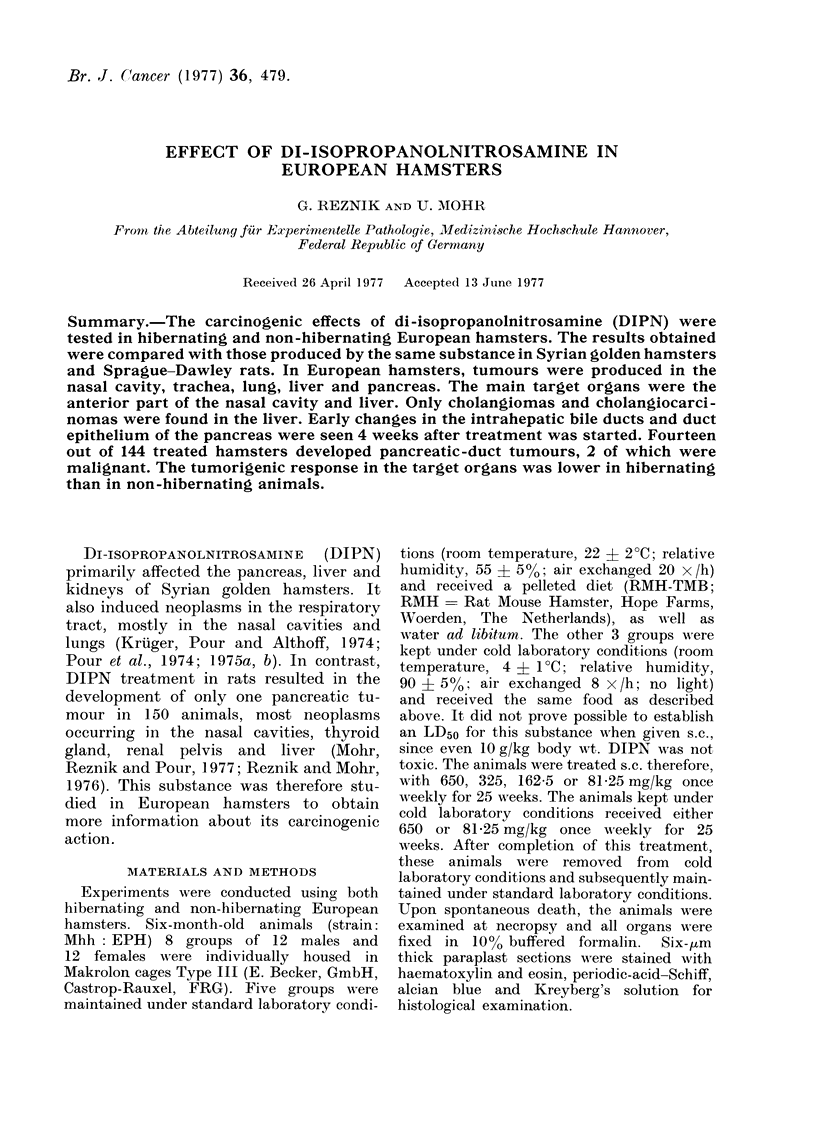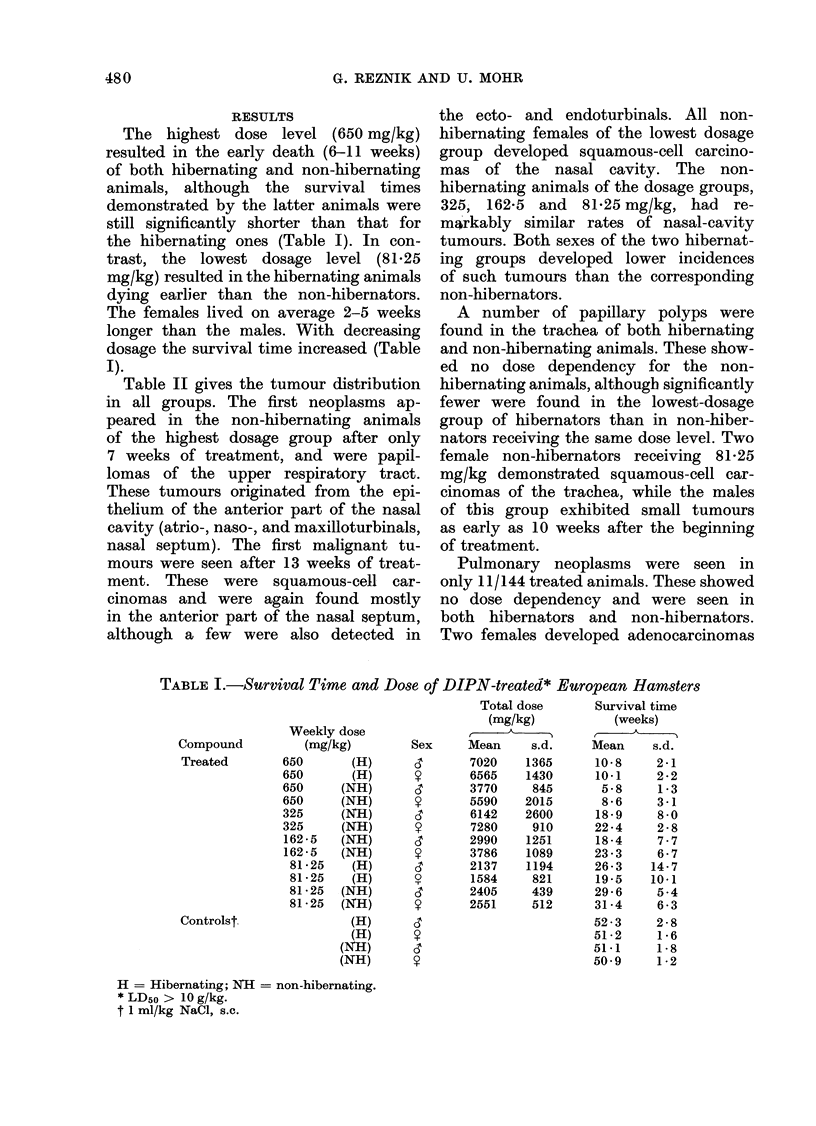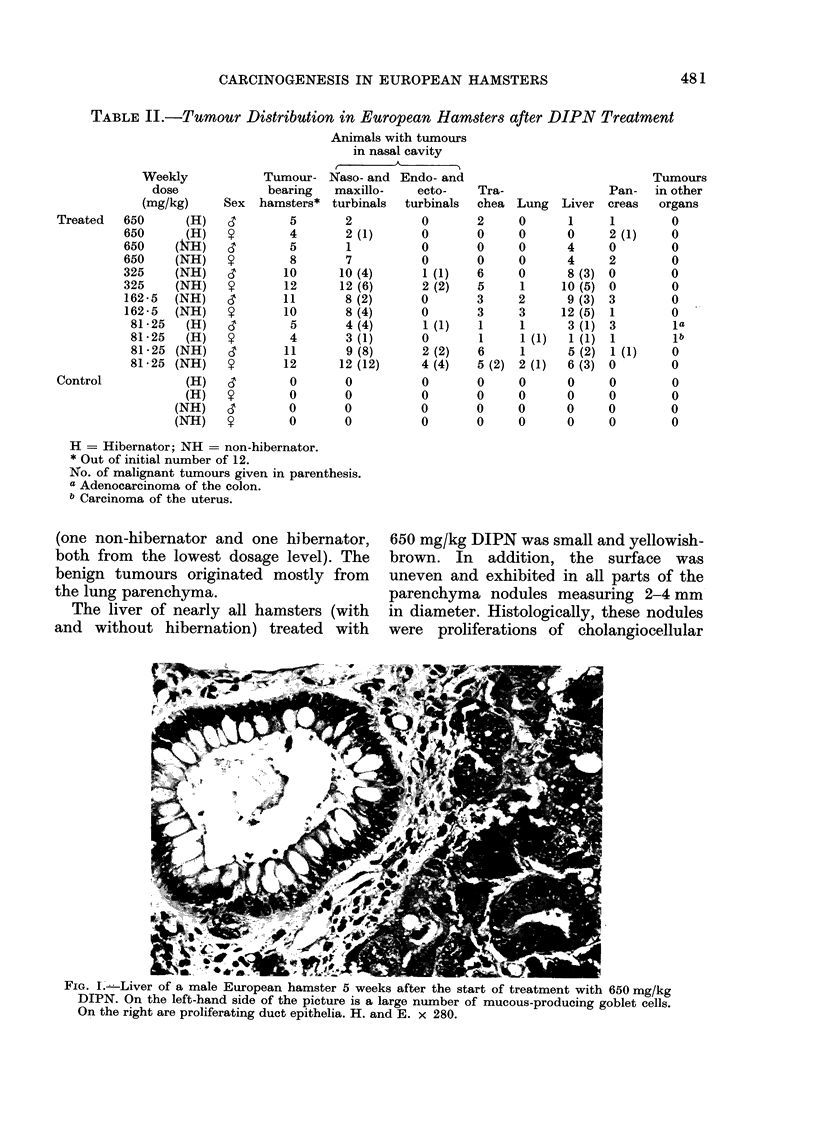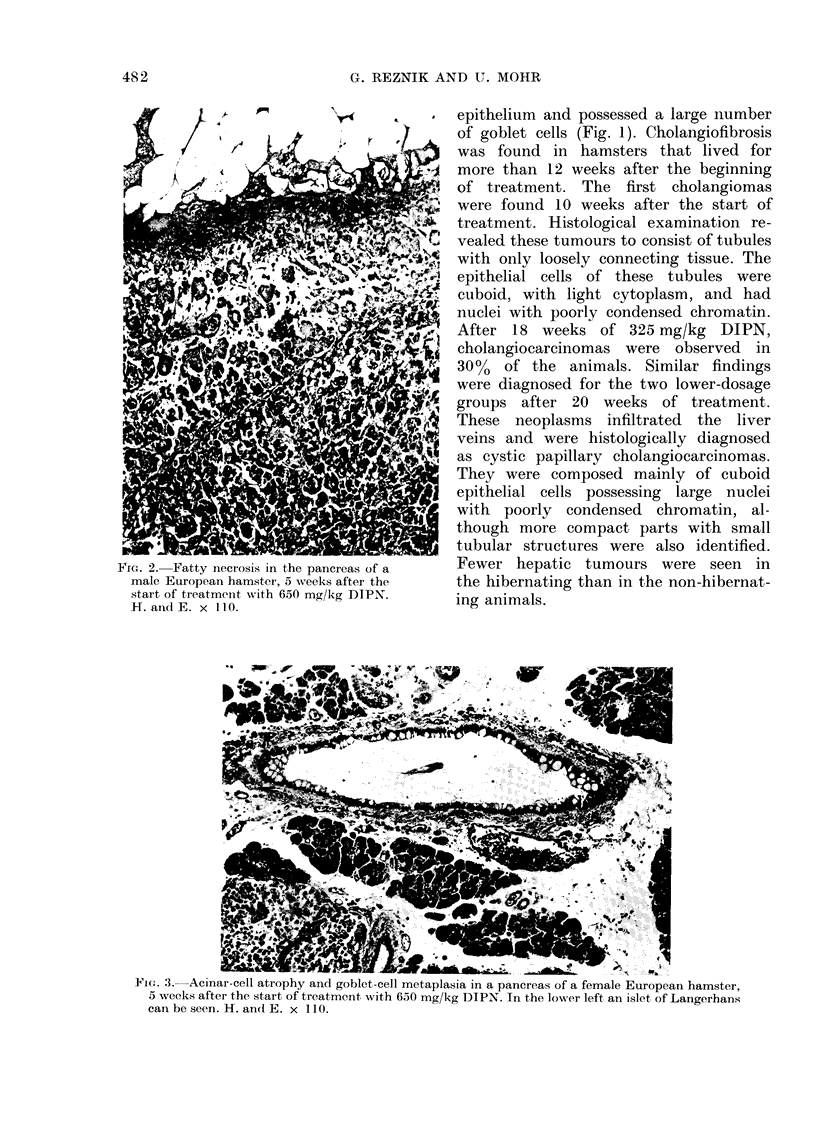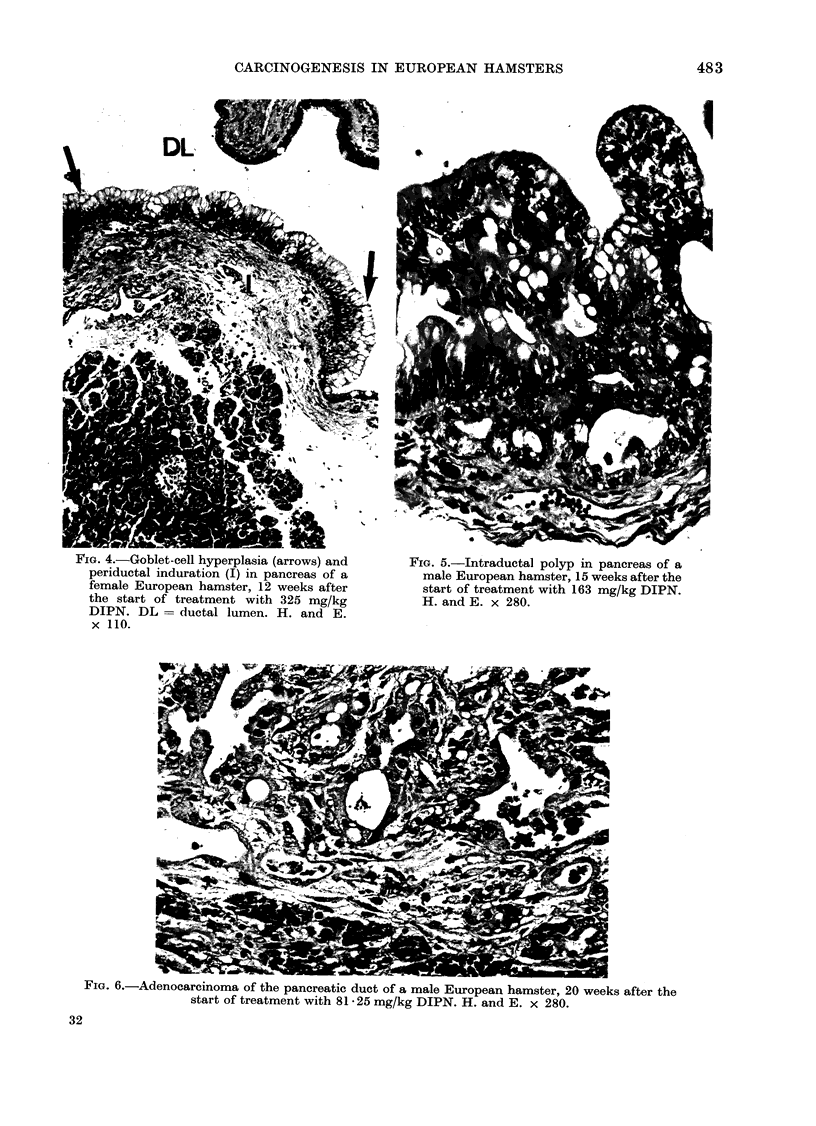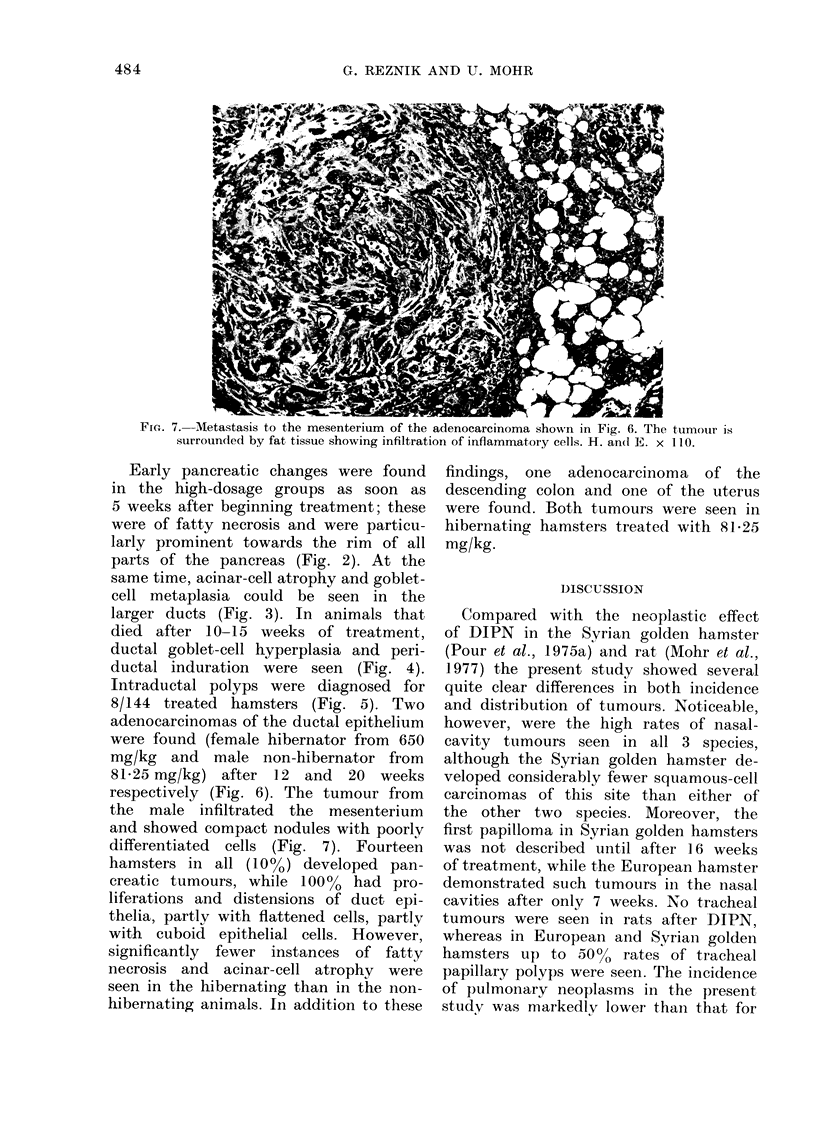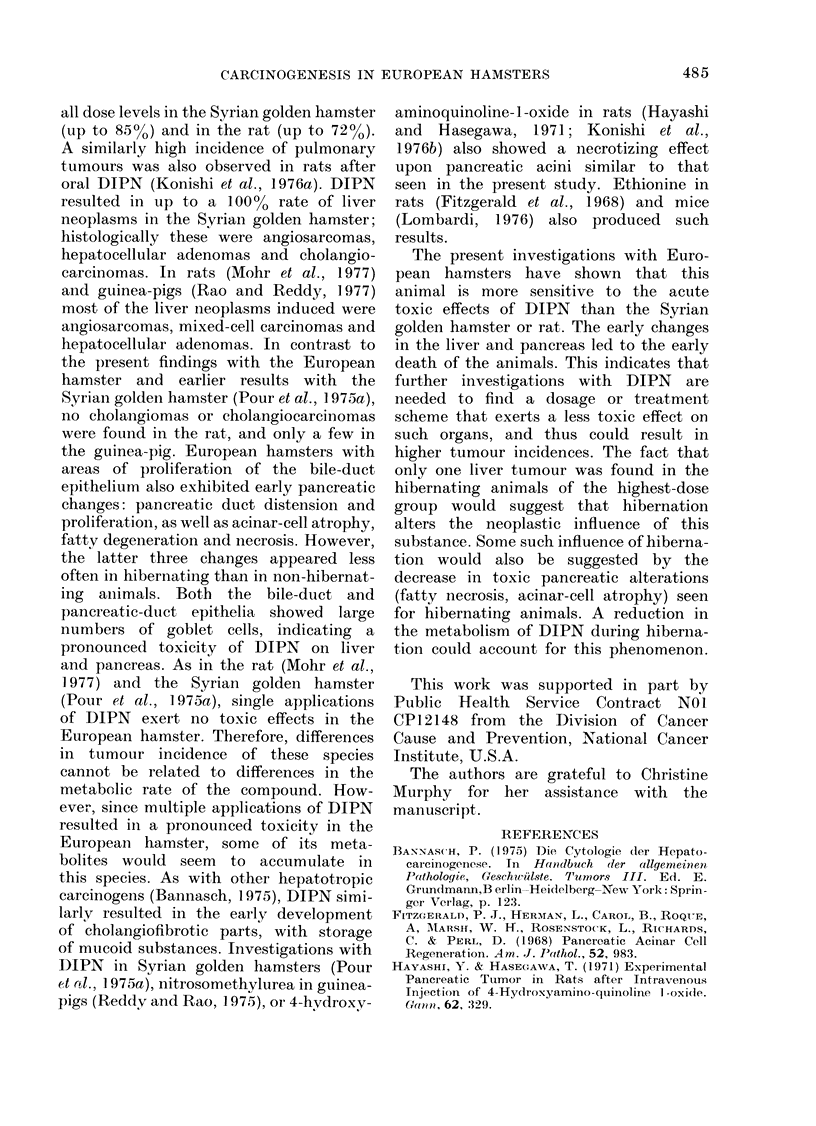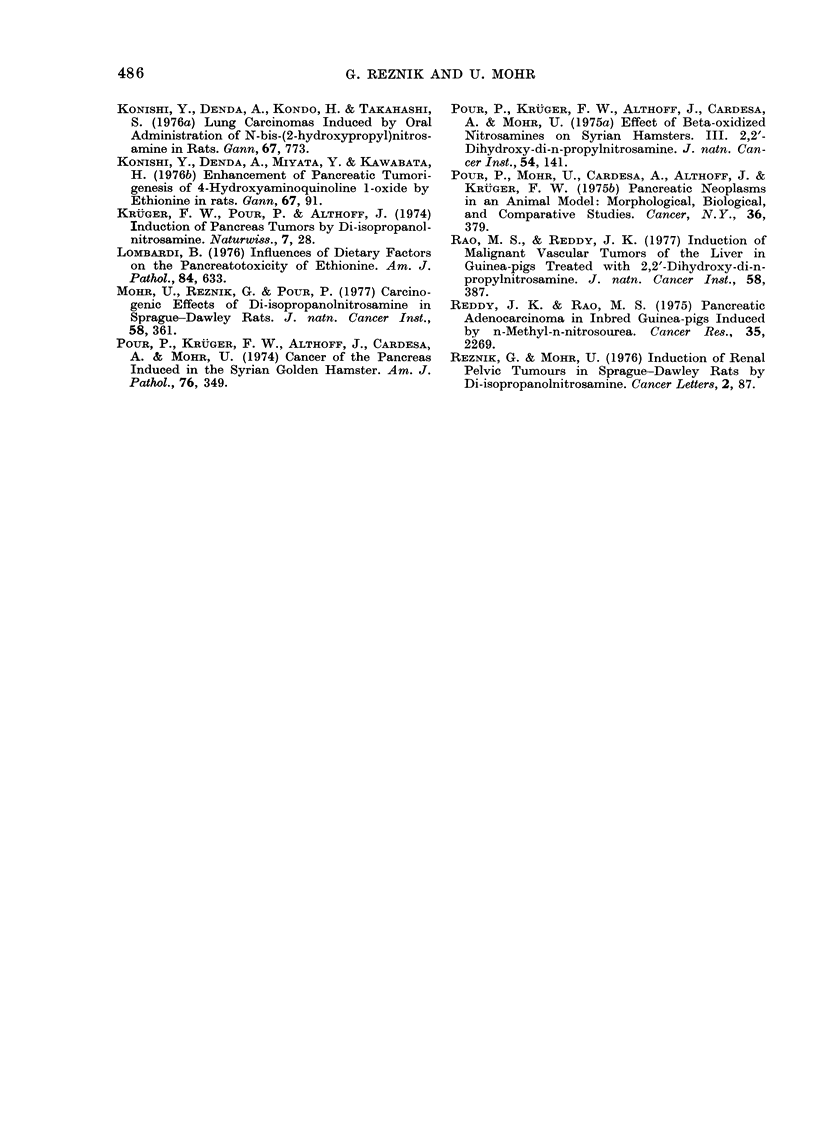# Effect of di-isopropanolnitrosamine in European hamsters.

**DOI:** 10.1038/bjc.1977.217

**Published:** 1977-10

**Authors:** G. Reznik, U. Mohr

## Abstract

**Images:**


*Br. J. Cancer* (1977) **36**, 479.

### EFFECT OF DI-ISOPROPANOLNITROSAMINE IN EUROPEAN HAMSTERS

G. REZNIK AND U. MOHR

*From the Abteilung für Experimentelle Pathologie, Medizinische Hochschule Hannover, Federal Republic of Germany*

Received 26 April 1977 Accepted 13 June 1977

**Summary.—The carcinogenic effects of di-isopropanolnitrosamine (DIPN) were tested in hibernating and non-hibernating European hamsters. The results obtained were compared with those produced by the same substance in Syrian golden hamsters and Sprague–Dawley rats. In European hamsters, tumours were produced in the nasal cavity, trachea, lung, liver and pancreas. The main target organs were the anterior part of the nasal cavity and liver. Only cholangiomas and cholangiocarcinomas were found in the liver. Early changes in the intrahepatic bile ducts and duct epithelium of the pancreas were seen 4 weeks after treatment was started. Fourteen out of 144 treated hamsters developed pancreatic-duct tumours, 2 of which were malignant. The tumorigenic response in the target organs was lower in hibernating than in non-hibernating animals.**

DI-ISOPROPANOLNITROSAMINE (DIPN) primarily affected the pancreas, liver and kidneys of Syrian golden hamsters. It also induced neoplasms in the respiratory tract, mostly in the nasal cavities and lungs (Krüger, Pour and Althoff, 1974; Pour *et al.*, 1974; 1975*a*, *b*). In contrast, DIPN treatment in rats resulted in the development of only one pancreatic tumour in 150 animals, most neoplasms occurring in the nasal cavities, thyroid gland, renal pelvis and liver (Mohr, Reznik and Pour, 1977; Reznik and Mohr, 1976). This substance was therefore studied in European hamsters to obtain more information about its carcinogenic action.

### MATERIALS AND METHODS

Experiments were conducted using both hibernating and non-hibernating European hamsters. Six-month-old animals (strain: Mhh : EPH) 8 groups of 12 males and 12 females were individually housed in Makrolon cages Type III (E. Becker, GmbH, Castrop-Rauxel, FRG). Five groups were maintained under standard laboratory conditions (room temperature, 22 $\pm$ 2°C; relative humidity, 55 $\pm$ 5%; air exchanged 20 $\times$/h) and received a pelleted diet (RMH-TMB; RMH = Rat Mouse Hamster, Hope Farms, Woerden, The Netherlands), as well as water *ad libitum*. The other 3 groups were kept under cold laboratory conditions (room temperature, 4 $\pm$ 1°C; relative humidity, 90 $\pm$ 5%; air exchanged 8 $\times$/h; no light) and received the same food as described above. It did not prove possible to establish an $LD_{50}$ for this substance when given s.c., since even 10 g/kg body wt. DIPN was not toxic. The animals were treated s.c. therefore, with 650, 325, 162·5 or 81·25 mg/kg once weekly for 25 weeks. The animals kept under cold laboratory conditions received either 650 or 81·25 mg/kg once weekly for 25 weeks. After completion of this treatment, these animals were removed from cold laboratory conditions and subsequently maintained under standard laboratory conditions. Upon spontaneous death, the animals were examined at necropsy and all organs were fixed in 10% buffered formalin. Six-$\mu$m thick paraplast sections were stained with haematoxylin and eosin, periodic-acid–Schiff, alcian blue and Kreyberg's solution for histological examination.

### RESULTS

The highest dose level (650 mg/kg) resulted in the early death (6–11 weeks) of both hibernating and non-hibernating animals, although the survival times demonstrated by the latter animals were still significantly shorter than that for the hibernating ones (Table I). In contrast, the lowest dosage level (81·25 mg/kg) resulted in the hibernating animals dying earlier than the non-hibernators. The females lived on average 2–5 weeks longer than the males. With decreasing dosage the survival time increased (Table I).

Table II gives the tumour distribution in all groups. The first neoplasms appeared in the non-hibernating animals of the highest dosage group after only 7 weeks of treatment, and were papillomas of the upper respiratory tract. These tumours originated from the epithelium of the anterior part of the nasal cavity (atrio-, naso-, and maxilloturbinals, nasal septum). The first malignant tumours were seen after 13 weeks of treatment. These were squamous-cell carcinomas and were again found mostly in the anterior part of the nasal septum, although a few were also detected in the ecto- and endoturbinals. All non-hibernating females of the lowest dosage group developed squamous-cell carcinomas of the nasal cavity. The non-hibernating animals of the dosage groups, 325, 162·5 and 81·25 mg/kg, had remarkably similar rates of nasal-cavity tumours. Both sexes of the two hibernating groups developed lower incidences of such tumours than the corresponding non-hibernators.

A number of papillary polyps were found in the trachea of both hibernating and non-hibernating animals. These showed no dose dependency for the non-hibernating animals, although significantly fewer were found in the lowest-dosage group of hibernators than in non-hibernators receiving the same dose level. Two female non-hibernators receiving 81·25 mg/kg demonstrated squamous-cell carcinomas of the trachea, while the males of this group exhibited small tumours as early as 10 weeks after the beginning of treatment.

Pulmonary neoplasms were seen in only 11/144 treated animals. These showed no dose dependency and were seen in both hibernators and non-hibernators. Two females developed adenocarcinomas

TABLE I.—*Survival Time and Dose of DIPN-treated* European Hamsters*

| Compound | Weekly dose (mg/kg) | | Sex | Total dose (mg/kg) | | Survival time (weeks) | |
|---|---|---|---|---|---|---|---|
| | | | | Mean | s.d. | Mean | s.d. |
| Treated | 650 | (H) | ♂ | 7020 | 1365 | 10·8 | 2·1 |
| | 650 | (H) | ♀ | 6565 | 1430 | 10·1 | 2·2 |
| | 650 | (NH) | ♂ | 3770 | 845 | 5·8 | 1·3 |
| | 650 | (NH) | ♀ | 5590 | 2015 | 8·6 | 3·1 |
| | 325 | (NH) | ♂ | 6142 | 2600 | 18·9 | 8·0 |
| | 325 | (NH) | ♀ | 7280 | 910 | 22·4 | 2·8 |
| | 162·5 | (NH) | ♂ | 2990 | 1251 | 18·4 | 7·7 |
| | 162·5 | (NH) | ♀ | 3786 | 1089 | 23·3 | 6·7 |
| | 81·25 | (H) | ♂ | 2137 | 1194 | 26·3 | 14·7 |
| | 81·25 | (H) | ♀ | 1584 | 821 | 19·5 | 10·1 |
| | 81·25 | (NH) | ♂ | 2405 | 439 | 29·6 | 5·4 |
| | 81·25 | (NH) | ♀ | 2551 | 512 | 31·4 | 6·3 |
| Controls† | | (H) | ♂ | | | 52·3 | 2·8 |
| | | (H) | ♀ | | | 51·2 | 1·6 |
| | | (NH) | ♂ | | | 51·1 | 1·8 |
| | | (NH) | ♀ | | | 50·9 | 1·2 |

H = Hibernating; NH = non-hibernating.
* $LD_{50} > 10$ g/kg.
† 1 ml/kg NaCl, s.c.

TABLE II.—*Tumour Distribution in European Hamsters after DIPN Treatment*

| | Weekly dose (mg/kg) | | Sex | Tumour-bearing hamsters* | Animals with tumours in nasal cavity: Naso- and maxillo-turbinals | Endo- and ecto-turbinals | Trachea | Lung | Liver | Pancreas | Tumours in other organs |
|---|---|---|---|---|---|---|---|---|---|---|---|
| Treated | 650 | (H) | ♂ | 5 | 2 | 0 | 2 | 0 | 1 | 1 | 0 |
| | 650 | (H) | ♀ | 4 | 2 (1) | 0 | 0 | 0 | 0 | 2 (1) | 0 |
| | 650 | (NH) | ♂ | 5 | 1 | 0 | 0 | 0 | 4 | 0 | 0 |
| | 650 | (NH) | ♀ | 8 | 7 | 0 | 0 | 0 | 4 | 2 | 0 |
| | 325 | (NH) | ♂ | 10 | 10 (4) | 1 (1) | 6 | 0 | 8 (3) | 0 | 0 |
| | 325 | (NH) | ♀ | 12 | 12 (6) | 2 (2) | 5 | 1 | 10 (5) | 0 | 0 |
| | 162·5 | (NH) | ♂ | 11 | 8 (2) | 0 | 3 | 2 | 9 (3) | 3 | 0 |
| | 162·5 | (NH) | ♀ | 10 | 8 (4) | 0 | 3 | 3 | 12 (5) | 1 | 0 |
| | 81·25 | (H) | ♂ | 5 | 4 (4) | 1 (1) | 1 | 1 | 3 (1) | 3 | 1[a] |
| | 81·25 | (H) | ♀ | 4 | 3 (1) | 0 | 1 | 1 (1) | 1 (1) | 1 | 1[b] |
| | 81·25 | (NH) | ♂ | 11 | 9 (8) | 2 (2) | 6 | 1 | 5 (2) | 1 (1) | 0 |
| | 81·25 | (NH) | ♀ | 12 | 12 (12) | 4 (4) | 5 (2) | 2 (1) | 6 (3) | 0 | 0 |
| Control | | (H) | ♂ | 0 | 0 | 0 | 0 | 0 | 0 | 0 | 0 |
| | | (H) | ♀ | 0 | 0 | 0 | 0 | 0 | 0 | 0 | 0 |
| | | (NH) | ♂ | 0 | 0 | 0 | 0 | 0 | 0 | 0 | 0 |
| | | (NH) | ♀ | 0 | 0 | 0 | 0 | 0 | 0 | 0 | 0 |

H = Hibernator; NH = non-hibernator.
* Out of initial number of 12.
No. of malignant tumours given in parenthesis.
[a] Adenocarcinoma of the colon.
[b] Carcinoma of the uterus.

(one non-hibernator and one hibernator, both from the lowest dosage level). The benign tumours originated mostly from the lung parenchyma.

The liver of nearly all hamsters (with and without hibernation) treated with 650 mg/kg DIPN was small and yellowish-brown. In addition, the surface was uneven and exhibited in all parts of the parenchyma nodules measuring 2–4 mm in diameter. Histologically, these nodules were proliferations of cholangiocellular

FIG. 1.—Liver of a male European hamster 5 weeks after the start of treatment with 650 mg/kg DIPN. On the left-hand side of the picture is a large number of mucous-producing goblet cells. On the right are proliferating duct epithelia. H. and E. × 280.

FIG. 2.—Fatty necrosis in the pancreas of a male European hamster, 5 weeks after the start of treatment with 650 mg/kg DIPN. H. and E. × 110.

epithelium and possessed a large number of goblet cells (Fig. 1). Cholangiofibrosis was found in hamsters that lived for more than 12 weeks after the beginning of treatment. The first cholangiomas were found 10 weeks after the start of treatment. Histological examination revealed these tumours to consist of tubules with only loosely connecting tissue. The epithelial cells of these tubules were cuboid, with light cytoplasm, and had nuclei with poorly condensed chromatin. After 18 weeks of 325 mg/kg DIPN, cholangiocarcinomas were observed in 30% of the animals. Similar findings were diagnosed for the two lower-dosage groups after 20 weeks of treatment. These neoplasms infiltrated the liver veins and were histologically diagnosed as cystic papillary cholangiocarcinomas. They were composed mainly of cuboid epithelial cells possessing large nuclei with poorly condensed chromatin, although more compact parts with small tubular structures were also identified. Fewer hepatic tumours were seen in the hibernating than in the non-hibernating animals.

FIG. 3.—Acinar-cell atrophy and goblet-cell metaplasia in a pancreas of a female European hamster, 5 weeks after the start of treatment with 650 mg/kg DIPN. In the lower left an islet of Langerhans can be seen. H. and E. × 110.

FIG. 4.—Goblet-cell hyperplasia (arrows) and periductal induration (I) in pancreas of a female European hamster, 12 weeks after the start of treatment with 325 mg/kg DIPN. DL = ductal lumen. H. and E. × 110.

FIG. 5.—Intraductal polyp in pancreas of a male European hamster, 15 weeks after the start of treatment with 163 mg/kg DIPN. H. and E. × 280.

FIG. 6.—Adenocarcinoma of the pancreatic duct of a male European hamster, 20 weeks after the start of treatment with 81·25 mg/kg DIPN. H. and E. × 280.

FIG. 7.—Metastasis to the mesenterium of the adenocarcinoma shown in Fig. 6. The tumour is surrounded by fat tissue showing infiltration of inflammatory cells. H. and E. × 110.

Early pancreatic changes were found in the high-dosage groups as soon as 5 weeks after beginning treatment; these were of fatty necrosis and were particularly prominent towards the rim of all parts of the pancreas (Fig. 2). At the same time, acinar-cell atrophy and goblet-cell metaplasia could be seen in the larger ducts (Fig. 3). In animals that died after 10–15 weeks of treatment, ductal goblet-cell hyperplasia and periductal induration were seen (Fig. 4). Intraductal polyps were diagnosed for 8/144 treated hamsters (Fig. 5). Two adenocarcinomas of the ductal epithelium were found (female hibernator from 650 mg/kg and male non-hibernator from 81·25 mg/kg) after 12 and 20 weeks respectively (Fig. 6). The tumour from the male infiltrated the mesenterium and showed compact nodules with poorly differentiated cells (Fig. 7). Fourteen hamsters in all (10%) developed pancreatic tumours, while 100% had proliferations and distensions of duct epithelia, partly with flattened cells, partly with cuboid epithelial cells. However, significantly fewer instances of fatty necrosis and acinar-cell atrophy were seen in the hibernating than in the non-hibernating animals. In addition to these findings, one adenocarcinoma of the descending colon and one of the uterus were found. Both tumours were seen in hibernating hamsters treated with 81·25 mg/kg.

### DISCUSSION

Compared with the neoplastic effect of DIPN in the Syrian golden hamster (Pour *et al.*, 1975a) and rat (Mohr *et al.*, 1977) the present study showed several quite clear differences in both incidence and distribution of tumours. Noticeable, however, were the high rates of nasal-cavity tumours seen in all 3 species, although the Syrian golden hamster developed considerably fewer squamous-cell carcinomas of this site than either of the other two species. Moreover, the first papilloma in Syrian golden hamsters was not described until after 16 weeks of treatment, while the European hamster demonstrated such tumours in the nasal cavities after only 7 weeks. No tracheal tumours were seen in rats after DIPN, whereas in European and Syrian golden hamsters up to 50% rates of tracheal papillary polyps were seen. The incidence of pulmonary neoplasms in the present study was markedly lower than that for all dose levels in the Syrian golden hamster (up to 85%) and in the rat (up to 72%). A similarly high incidence of pulmonary tumours was also observed in rats after oral DIPN (Konishi *et al.*, 1976*a*). DIPN resulted in up to a 100% rate of liver neoplasms in the Syrian golden hamster; histologically these were angiosarcomas, hepatocellular adenomas and cholangiocarcinomas. In rats (Mohr *et al.*, 1977) and guinea-pigs (Rao and Reddy, 1977) most of the liver neoplasms induced were angiosarcomas, mixed-cell carcinomas and hepatocellular adenomas. In contrast to the present findings with the European hamster and earlier results with the Syrian golden hamster (Pour *et al.*, 1975*a*), no cholangiomas or cholangiocarcinomas were found in the rat, and only a few in the guinea-pig. European hamsters with areas of proliferation of the bile-duct epithelium also exhibited early pancreatic changes: pancreatic duct distension and proliferation, as well as acinar-cell atrophy, fatty degeneration and necrosis. However, the latter three changes appeared less often in hibernating than in non-hibernating animals. Both the bile-duct and pancreatic-duct epithelia showed large numbers of goblet cells, indicating a pronounced toxicity of DIPN on liver and pancreas. As in the rat (Mohr *et al.*, 1977) and the Syrian golden hamster (Pour *et al.*, 1975*a*), single applications of DIPN exert no toxic effects in the European hamster. Therefore, differences in tumour incidence of these species cannot be related to differences in the metabolic rate of the compound. However, since multiple applications of DIPN resulted in a pronounced toxicity in the European hamster, some of its metabolites would seem to accumulate in this species. As with other hepatotropic carcinogens (Bannasch, 1975), DIPN similarly resulted in the early development of cholangiofibrotic parts, with storage of mucoid substances. Investigations with DIPN in Syrian golden hamsters (Pour *et al.*, 1975*a*), nitrosomethylurea in guinea-pigs (Reddy and Rao, 1975), or 4-hydroxyaminoquinoline-1-oxide in rats (Hayashi and Hasegawa, 1971; Konishi *et al.*, 1976*b*) also showed a necrotizing effect upon pancreatic acini similar to that seen in the present study. Ethionine in rats (Fitzgerald *et al.*, 1968) and mice (Lombardi, 1976) also produced such results.

The present investigations with European hamsters have shown that this animal is more sensitive to the acute toxic effects of DIPN than the Syrian golden hamster or rat. The early changes in the liver and pancreas led to the early death of the animals. This indicates that further investigations with DIPN are needed to find a dosage or treatment scheme that exerts a less toxic effect on such organs, and thus could result in higher tumour incidences. The fact that only one liver tumour was found in the hibernating animals of the highest-dose group would suggest that hibernation alters the neoplastic influence of this substance. Some such influence of hibernation would also be suggested by the decrease in toxic pancreatic alterations (fatty necrosis, acinar-cell atrophy) seen for hibernating animals. A reduction in the metabolism of DIPN during hibernation could account for this phenomenon.

This work was supported in part by Public Health Service Contract N01 CP12148 from the Division of Cancer Cause and Prevention, National Cancer Institute, U.S.A.

The authors are grateful to Christine Murphy for her assistance with the manuscript.

### REFERENCES

BANNASCH, P. (1975) Die Cytologie der Hepatocarcinogenese. In *Handbuch der allgemeinen Pathologie, Geschwülste. Tumors III.* Ed. E. Grundmann, Berlin–Heidelberg–New York: Springer Verlag, p. 123.

FITZGERALD, P. J., HERMAN, L., CAROL, B., ROQUE, A, MARSH, W. H., ROSENSTOCK, L., RICHARDS, C. & PERL, D. (1968) Pancreatic Acinar Cell Regeneration. *Am. J. Pathol.*, **52**, 983.

HAYASHI, Y. & HASEGAWA, T. (1971) Experimental Pancreatic Tumor in Rats after Intravenous Injection of 4-Hydroxyamino-quinoline 1-oxide. *Gann*, **62**, 329.

KONISHI, Y., DENDA, A., KONDO, H. & TAKAHASHI, S. (1976*a*) Lung Carcinomas Induced by Oral Administration of N-bis-(2-hydroxypropyl)nitrosamine in Rats. *Gann*, **67**, 773.

KONISHI, Y., DENDA, A., MIYATA, Y. & KAWABATA, H. (1976*b*) Enhancement of Pancreatic Tumorigenesis of 4-Hydroxyaminoquinoline 1-oxide by Ethionine in rats. *Gann*, **67**, 91.

KRÜGER, F. W., POUR, P. & ALTHOFF, J. (1974) Induction of Pancreas Tumors by Di-isopropanolnitrosamine. *Naturwiss.*, **7**, 28.

LOMBARDI, B. (1976) Influences of Dietary Factors on the Pancreatotoxicity of Ethionine. *Am. J. Pathol.*, **84**, 633.

MOHR, U., REZNIK, G. & POUR, P. (1977) Carcinogenic Effects of Di-isopropanolnitrosamine in Sprague–Dawley Rats. *J. natn. Cancer Inst.*, **58**, 361.

POUR, P., KRÜGER, F. W., ALTHOFF, J., CARDESA, A. & MOHR, U. (1974) Cancer of the Pancreas Induced in the Syrian Golden Hamster. *Am. J. Pathol.*, **76**, 349.

POUR, P., KRÜGER, F. W., ALTHOFF, J., CARDESA, A. & MOHR, U. (1975*a*) Effect of Beta-oxidized Nitrosamines on Syrian Hamsters. III. 2,2′-Dihydroxy-di-n-propylnitrosamine. *J. natn. Cancer Inst.*, **54**, 141.

POUR, P., MOHR, U., CARDESA, A., ALTHOFF, J. & KRÜGER, F. W. (1975*b*) Pancreatic Neoplasms in an Animal Model: Morphological, Biological, and Comparative Studies. *Cancer, N.Y.*, **36**, 379.

RAO, M. S., & REDDY, J. K. (1977) Induction of Malignant Vascular Tumors of the Liver in Guinea-pigs Treated with 2,2′-Dihydroxy-di-n-propylnitrosamine. *J. natn. Cancer Inst.*, **58**, 387.

REDDY, J. K. & RAO, M. S. (1975) Pancreatic Adenocarcinoma in Inbred Guinea-pigs Induced by n-Methyl-n-nitrosourea. *Cancer Res.*, **35**, 2269.

REZNIK, G. & MOHR, U. (1976) Induction of Renal Pelvic Tumours in Sprague–Dawley Rats by Di-isopropanolnitrosamine. *Cancer Letters*, **2**, 87.